# A Significant Change in Free Amino Acids of Soybean (*Glycine max* L. Merr) through Ethylene Application

**DOI:** 10.3390/molecules26041128

**Published:** 2021-02-20

**Authors:** Yeong Jun Ban, Yeong Hun Song, Jeong Yoon Kim, Joon Yung Cha, Imdad Ali, Aizhamal Baiseitova, Abdul Bari Shah, Woe-Yeon Kim, Ki Hun Park

**Affiliations:** Division of Applied Life Science (BK21 plus), IALS, RILS, Gyeongsang National University, Jinju 52828, Korea; banyoung972@naver.com (Y.J.B.); yh0126@hanmail.net (Y.H.S.); foryou6633@gmail.com (J.Y.K.); jycha@gnu.ac.kr (J.Y.C.); imdad_takkar@yahoo.com (I.A.); aizhabaiseitova@gmail.com (A.B.); abs_korea28@gnu.ac.kr (A.B.S.); kim1312@gnu.ac.kr (W.-Y.K.)

**Keywords:** free amino acids, soybean, ethylene, asparagine, gas chromatography–mass spectrometry (GC-MS)

## Abstract

In this study, the changes in free amino acids of soybean leaves after ethylene application were characterized based on quantitative and metabolomic analyses. All essential and nonessential amino acids in soybean leaves were enhanced by fivefold (250 to 1284 mg/100 g) and sixfold (544 to 3478 mg/100 g), respectively, via ethylene application. In particular, it was found that asparagine is the main component, comprising approximately 41% of the total amino acids with a twenty-five fold increase (78 to 1971 mg/100 g). Moreover, arginine and branched chain amino acids (Val, Leu, and Ile) increased by about 14 and 2–5 times, respectively. The increase in free amino acid in stem was also similar to the leaves. The metabolites in treated and untreated soybean leaves were systematically identified by gas chromatography–mass spectrometry (GC-MS), and partial variance discriminant analysis (PLS-DA) scores and heat map analysis were given to understand the changes of each metabolite. The application of ethylene may provide good nutrient potential for soybean leaves.

## 1. Introduction

Amino acids are the basic units of protein and have important functions in various physiological processes (such as skeletal muscle function) and pathophysiological conditions (such as atrophy, and sarcopenia) [1,2]. They are also associated with numerous biological benefits, such as anti-inflammatory and antibacterial effects, in addition to contributing to vital processes like hormones synthesis and neurotransmitters [3,4]. Some amino acids may also be taken in supplement form for a natural way to boost athletic performance [5]. For a specific example, branched-chain amino acids (Leu, Ile, and Val) have been shown to accelerate recovery from muscle damage and fatigue after exercise [6]. Arginine is critically important for T-cell proliferation and acts as a source for nitric oxide (NO), which is key for host immune response and defenses [7,8]. Asparagine increases the resistance to fatigue and improves the smooth functioning of the liver [9]. Asparagine is also known for its key role in the biosynthesis of glycoprotein [10]. Particularly, free amino acids (FAAs) might be superior to protein as a dietary source because FAAs are absorbed faster and assimilated more efficiently than more complex forms such as peptides and proteins [11].

Ethylene is a phytohormone induced by various factors such as microbial pathogens, insect attacks, and stimuli. This phytohormone regulates a wide range of physiological processes, including fruit ripening and metabolite pathway activation [12,13]. Recent literature has proven that soybean proteins provide health benefits, such as blood cholesterol reduction, protection against coronary heart disease, and anti-obesity properties [14,15].

Soybean (*Glycine max* L. Merr) leaves are attractive nutraceutical sources because of their biological benefits to human chronic diseases. Their extracts have demonstrated anti-obesity, antidiabetic, antiatherogenic, and anti-inflammatory effects [16,17]. Coumarol, the main metabolite in soybean leaves, has shown significant α-glucosidase inhibitory effects and induces cancer cell senescence [18,19]. Yellow leaf extract was effective at suppressing hyperglycemia and hepatic steatosis through enhancing adiponectin-receptor signaling AMPK (AMP-activated protein kinase) activation and pancreatic β-cell functionality [20]. More than 36 phenolic compounds from soybean leaves have been reported, and the main bioactive components are flavonol, isoflavone, pterocarpan, and soyasaponin derivatives [21,22]. Secondary metabolites in the leaves were associated with the growth stage. Pterocarpan derivatives especially started to appear at the R2 stage and were optimized at the R7 stage [23]. Moreover, ethylene application on soybean leaves led to a significant accumulation of phytoestrogens (daidzein, genistein, malonyldaidzein, and malonylgenistin) [24,25]. It is generally accepted that the formation of free amino acids at the ripening stage leads to an increase in ethylene. It was recently reported that ethylene treatment of green papayas led to a change in some amino acids and fatty acids during the ripening process [26].

The objective of this study was to examine the influence of ethylene application on FAAs in soybean leaves, as well as the plant’s asparagine biosynthetic pathway. Quantitative and metabolomic analyses were carried out to illustrate the relationship between ethylene treated and untreated samples by using amino acid analyzer and gas chromatography–mass spectrometry (GC-MS). Multivariate statistical analysis was done to illustrate the action of ethylene on the asparagine biosynthetic pathway. To the best of our knowledge, there have been no reports of treating ethylene to increase the amount of free amino acids.

## 2. Results and Discussion

### 2.1. Changes in Free Amino Acids after Ethylene Application

**Figure 1:**
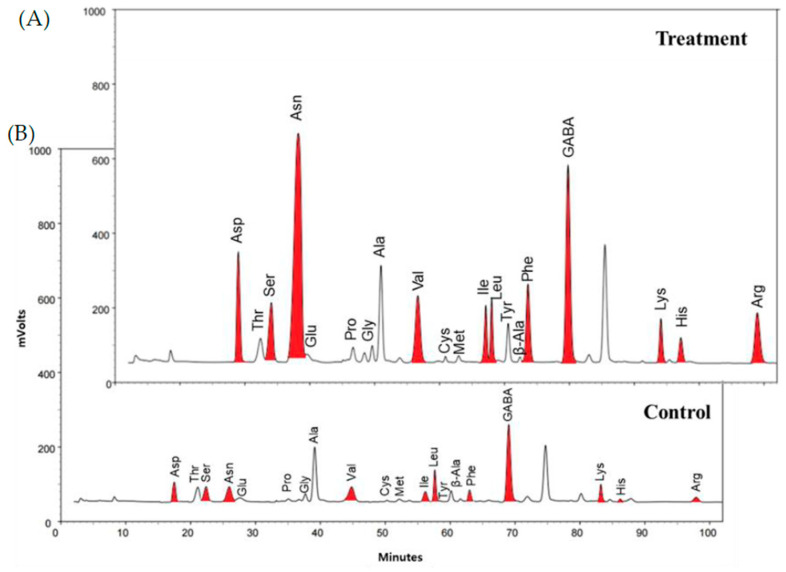
The chromatographic profiles from amino acid analysis of soybean leaves: (**A**) ethylene treatment and (**B**) untreated (control).

This study observed that the application of ethylene resulted in changes in free amino acids (FAAs) in soybean leaves. The chromatographic profiles of FAAs showed significant changes between the ethylene treated and untreated samples (Figure 1A,B). The most changed FAAs, such as aspartic acid (Asp), serine (Ser), asparagine (Asn), valine (Val), isoleucine (Ile), leucine (Leu), phenylalanine (Phe), γ-aminobutanoic acid (GABA), lysine (Lys), histidine (His), and arginine (Arg), were colored with red.

The quantitative results illustrated that the essential and non-essential FAAs in soybean leaves increased by fivefold (250 to 1284 mg/100 g) and sixfold (544 to 3478 mg/100 g), respectively. Asn was the most changed FAA, increasing by sixfold, from 78 mg to 1971 mg/100 g. Furthermore, ethylene made Asn the most abundant FAA carrier, while previously it was a minor carrier. In particular, due to the health benefits associated with anti-fatigue and central nervous system balance, Asn-rich plants may have the potential to be used in functional foods [27]. Remarkable variations were also observed in the remaining component. In other words, ethylene treatment resulted in a two–sevenfold increase in Val, Ile, and Leu. It is well documented that these branched chain amino acids have a vital biological function that promotes wound healing, stimulates insulin production, and attenuates fatigue [28,29]. Ethylene enhanced Asp eightfold, which promotes hormone production and releases and normalizes the nervous system [30]. It especially influenced arginine production, which increased by fourteenfold. Arginine boosts nitric oxide production and relaxes blood vessels [31]. The decarboxylated product of glutamic acid, GABA, supports neurotransmitters in the brain [32]. 

GABA increased up to 439 mg/100 g from 199 mg/100 g after ethylene treatment (Table 1). This phenomenon suggests that ethylene may influence the signal transduction and recognition pathway throughout the soybean plant’s development stage [33].

The changes in nitrogen level were estimated to show whether the increase in free amino acids is due to protein degradation. Total nitrogen level was increased from 2.87 g/100 g to 3.68 g/100 g to rationalized that ethylene associated with amino acid synthesis.

FAA stem compositions showed similar patterns to leaves after ethylene application. Ethylene led to a significant increase in essential FAAs (threefold, 164 mg to 611 mg/100 g) and non-essential FAAs (fivefold, 774 mg to 4124 mg/100 g) (Table 1). Similar to the results found in the leaves, Asn became the most changed FAA after ethylene treatment, turning into the most abundant FAA with 362 to 2975 mg/100 g (Table 1). Overall, ethylene application led to distinctive changes in FAA in soybean leaves and stems. To the best of our knowledge, the current research is the first to demonstrate the FAA contents in soybean plants through ethylene action.

### 2.2. Metabolomic Analysis via GC-MS

**Figure 2:**
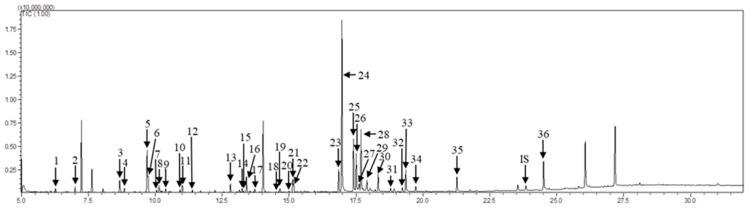
Quality control BPI chromatogram of ethylene untreated and treated soybean leaves extracts using GC-MS. **1**, lactic acid; **2**, alanine; **3**, malonic acid; **4**, valine; **5**, phosphoric acid; **6**, glycerol; **7**, isoleucine; **8**, proline; **9**, succinic acid; **10**, fumaric acid; **11**, serine; **12**, threonine; 13, malic acid; **14**, aspartic acid; **15**, oxoproline; 16, 4-aminobutanoic acid; **17**, threonic acid; **18**, glutamic acid; **19**, phenylalnine; **20**, arabinose; **21**, asparagine; **22**, lyxose; **23**, citric acid; **24**, pinitol; **25**, fructose; 26, fructose; **27**, galactose; **28**, mannose; **29**, glucose; **30**, inositol; **31**, gluconic acid; **32**, galactaric acid; **33**, palmitic acid; **34**, myo-inositol; **35**, stearic acid; **36**, sucrose.

The metabolites in the treated and untreated soybean leaves were systematically identified using a GC-MS-based metabolomic technique. Metabolites, including free amino acids, sugars, and organic acids were extracted with water from soybean leaves. The water extract was derivatized with *N*,*O*-bis(trimethylsilyl)trifluoroacetamide (BSTFA) and trimethylchlorosilane (TMSC) and then applied to GC-MS. A representative base peak intensity (BPI) chromatogram in Figure 2 was a quality control (QC) chromatogram of ethylene-untreated and treated soybean leaves extracts using GC-MS. In total, 36 metabolites were found after statistical analysis of the normalized metabolites using ANOVA with Duncan’s test (*p* < 0.05). The identified metabolites, consisting of amino acids (Ala, Val, Ile, Pro, Ser, Thr, Asp, GABA, Glu, Phe, Asn, and oxoproline), organic acids (lactate, malonate, phosphoric aicd, succinate, fumarate, malate, threonate, citrate, gluconate and galactonate), sugars (arabinose, lyxose, fructose, galactose, mannose, glucose, pinitol, inositol, myo-inositol, and sucrose), fatty acids (palmitate and stearate), and lipid metabolite (glycerol), are shown in Figure 2 and the identification of metabolites contributing to the separation among sample groups is illustrated in Table S1.

**Figure 3:**
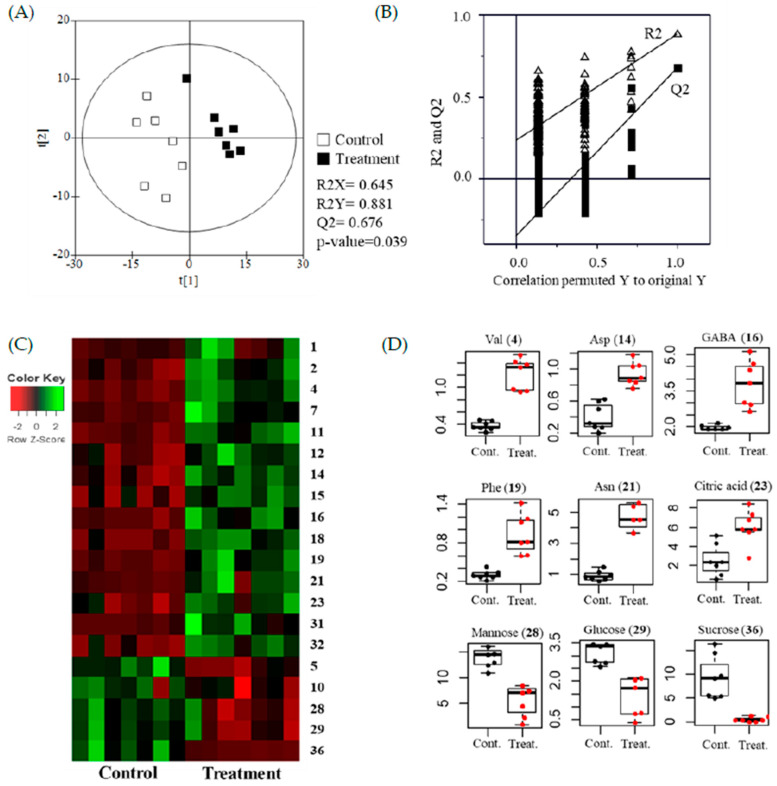
Metabolomic analysis of soybean leaves using gas chromatography–mass spectrometry (GC-MS). (**A**) Partial variance discriminant analysis (PLS-DA) plots of untreated (control) and ethylene treated (treatment). (**B**) Performance of the permutation tests validated from the PLS-DA model. (**C**) Heatmap for the 20 metabolites (*p* < 0.05) between control and treatment. (**D**) Boxplots of representative metabolites.

The results were statistically analyzed using the PLS-DA. Modeling quality parameters were validated for fitness and predictability as follows: R2X = 0.645, R2Y = 0.789, and Q2 = 0.676 (Figure 3A,B). Values were obtained from cross validation by a permutation (R2 intercept = 0.212, Q2 intercept = −0.353, and *p* value = 0.039), which indicated that PLS-DA plots were statistically acceptable (see: supplementary materials). In addition, the QC data were closely clustered in the PLS-DA score plots, indicating that the data quality was acceptable for metabolomics research. The PLS-DA score plots were significantly distinguished between treated and untreated samples (Figure 3A).

The glycolysis and TCA (tricarboxylic acid) cycle supply carbons for amino acid biosynthesis. Amino acids can be synthesized from pyruvate in the glycolysis process, and oxaloacetate and α-ketoglutarate in the TCA cycle [34]. According to the constructed corresponding heatmap and boxplots (Figure 3C,D), as well as each coordinate’s calculated VIP value (>1), the GC metabolites during ethylene application were determined. The results showed that the increased levels of all amino acids were coupled with decreased levels of sugars (fructose, mannose, glucose, and sucrose), which were the sources of the glycolysis process and TCA cycle. Citrate was the starting substance in the TCA cycle and it increased significantly. Phosphorylation was the source in sugars, causing phosphoric acid to decrease. Many studies have clearly show that signaling molecules (such as ethylene) stimulate the biosynthetic pathways of metabolites supported by the TCA cycle [35,36]. Recently, a multi-omics analysis showed that ethylene might activate many physiological pathways in soybean leaves, including the TCA cycle and isoflavone biosynthesis [37].

## 3. Materials and Methods

### 3.1. Plant Material and Experiment Design

Soybean (*Glycine max* L. Merr) was cultivated in greenhouse pots over a period of about 60 days until the plant reached a maximum growth stage, R1, which was the beginning of flowering to full bloom. About 60 soybean pots in the growth stage R1 were put in the chamber. The chamber was designed to be air-sealed in order to maintain the ethylene concentration and control humidity and temperature. The ethylene concentrations were measured using a portable measuring sensor (COSMOS, P-3160, Tokyo, Japan). Subsequently, the chamber was maintained for 36 h in the following conditions: 2500 µg/g concentration of ethylene, 70% relative humidity, and 35 °C. After 36 h, plant leaves were randomly collected from each pot, with three replicates (for quantitative analysis) and seven replicates (for metabolomics analysis). They were then cut into small pieces using a laboratory blade cutter, dried at 35 °C, and used for further analysis.

### 3.2. Quantitative Analysis of Free Amino Acids Using HPLC

FAAs were determined with a slightly modified version of the reported method [38]. The milled samples (each 100 mg) were extracted with 5 mL deionized water at 60 °C for 1 h. Protein and peptides were precipitated by adding 1 mL of 10% (*v*/*v*) sulfosalicylic acid. The solvent was centrifuged for 30 min at 5000 rpm, and supernatant was then filtered using a 0.45 µm filter. The solvent was removed using a vacuum evaporator at 45 °C. The concentrate was finally dissolved in 2 mL of 0.2 M lithium citrate buffer, pH 2.2 (Biochrom Ltd., Cambridge, UK), and filtered through a 0.45 µm filter. The obtained samples were analyzed in 48 h and stored at 4 °C until analysis.

About 20 μL of this mixture was injected into a Biochrom + 30 system (Biochrom Ltd., Cambridge, UK), equipped with a high-pressure PEEK (Poly. Ethyl Ketone) column packed with Ultropac 8 cation exchange resin. Amino acids were eluted with varying temperature, ionic strength, and pH of the lithium citrate buffer, according to the instructions given by the manufacturer of the instruments. Detection was carried out using the reaction of ninhydrin with amino acids that formed colored compounds, and was then photometrically detected at 440 nm (primary amines) and 570 nm (secondary amines). BioSys control software and EZChrom Elite data handling software (Biochrom Ltd., Cambridge, UK) were used for data acquisition and analysis. Amino acid calibration was prepared at known concentrations for each analyte with commercially existing standards (Sigma-Aldrich, St. Louis, MO, USA) and was run each time with freshly prepared ninhydrin. Each amino acid was identified and quantified on the basis of its external calibration curve (linearity of R2 > 0.995).

### 3.3. Metabolite Extraction and Derivatization for GC-MS Analysis

For GC-MS analysis of soybean plants, the milled samples (each 100 mg) were extracted with 5 mL of water at 60 °C for 1 h. The 30 µL of supernatant was dried using a vacuum CentriVap concentrator (Labconco, Kansas City, MO, USA) at 40 °C. The dried samples were dissolved in 2% methoxyamine hydrochloride in 20 mg/mL pyridine, with dicyclohexyl phthalate as an internal standard. They were then incubated at 37 °C for 90 min followed by derivatization in 80 µL *N*,*O*-bis (trimethylsilyl)trifluoroacetamide (BSTFA) and 1% trimethylchlorosilane (TMCS) at 70 °C for 30 min. This was then analyzed via GC-MS [39].

### 3.4. GC-MS Analysis

The derivatized water extracts were examined using a Shimadzu GC-2010 plus (Tokyo, Japan) provided with a DB-5 ms capillary column (30 m × 0.25 mm, 0.25 μm, Agilent J-W, Santa Clara, CA, USA). One microliter of water extract was injected into the capillary column with a split ratio of 1:40. Helium was used as a carrier gas at a flow rate of 1 mL/min, and the injection temperature was set to 200 °C. The oven temperature was set from 70 to 210 °C at a rate of 7 °C/min with initial and final holding times of 2 min, and from 210 to 320 °C at a rate of 10 °C/min with the 7 min holding time. The GC column effluent was detected with a Shimadzu GC-MS-TQ 8030 mass spectrometer (Tokyo, Japan) in electron ionization (EI) mode (70 eV). The temperatures of the source and interface were 230 and 280 °C, respectively. The MS spectra were monitored in the full scan mode from *m*/*z* 45–800 with a scan event time of 0.3 s and a scan speed of 2000 μ/s.

### 3.5. Data Processing

MS data, including a deconvolution of mass spectra, data collection, alignment, and normalization, were analyzed using GC-MS and processed using a PostRun analysis (Shimadzu, Tokyo, Japan). Metabolite peaks were deconvoluted and collected using an area threshold of 4000. To normalize all MS data, an internal standard of dicyclohexyl phthalate was used. Identification of metabolites was carried out by comparing their mass spectra and retention indices (RI), which were obtained using a number of n-alkanes (C8–C40) with the Wiley and NIST mass spectral databases, the published RIs, and authentic standards.

### 3.6. Statistical Analysis

GC-MS data sets, processed with Analyzer Pro, were subjected to multivariate statistical analysis with SIMCA-P+ version 12.0.1 (Umetrics, Umeå, Sweden). The visualization among the sample groups was done using the partial square discrimination analysis (PLS-DA). To statistically analyze the differences between the groups, the Hotelling’s T2 test was used. Distant samples in the ellipse region, defined as having a 95% confidence interval of the simulated changes, were excluded from further analysis. Evaluation of the PLS-DA model quality was done using the goodness of fit measure (R2X and R2Y) and predictive ability (Q2Y). It was validated using sevenfold cross-validation with a permutation test. Metabolites contributed to the discrimination between groups were established and identified on the basis of a variable importance in the projection (VIP) value > 1, calculated by the PLS-DA and one-way analysis of variance (ANOVA) with Duncan’s test (*p* < 0.05) using SPSS 17.0 (SPSS Inc., Chicago, IL, USA). A heat map (drawn by R with gplots) was used to visualize the identified metabolites with significant differences (*p* < 0.05), which represents the z-score transformed data of metabolites. The green–red color scale was used to construct the heat map to indicate the increase and decrease of metabolite levels.

## 4. Conclusions

This study reported that the application of ethylene resulted in a significant increase in free amino acids, which could improve the nutritional quality of soybean leaves. All es-sential and non-essential amino acids of soybean leaves were enhanced fivefold (250 to 1284 mg/100 g) and sixfold (544 to 3478 mg/100 g), respectively. Interestingly, asparagine (Asn) was found to be the most changed FAA, showing a twenty-five fold increase (78 to 1971 mg/100 g), accounting for 41% of the total FAA. The remaining valuable essential FAAs included arginine and branched chain FAAs (Val, Leu, and Ile), both of which distinctively increased. The low molecular metabolites in the treated and untreated soybean leaves were systematically identified using GC-MS to give successfully separated PLS-DA scores and to obtain information regarding energy production. Our results showed that ethylene application might improve the nutraceutical potential of soybean leaves.

## Figures and Tables

**Table 1:** Effects of ethylene treatment on free amino acid contents (mg/100 g) in soybean leaves and stems.

Amino Acids	Leaves		Stems	
	Control	Treatment	Control	Treatment
NEAA ^a^ Asp Ser Asn Glu Pro Gly Ala Cys Tyr β-Ala GABA Total	34 ± 3.5 d 27 ± 5.2 c 78 ± 15 d 21 ± 1.4 c 40 ± 0.7 d 10 ± 0.8 c 85 ± 3.2 b 4 ± 0.5 b 34 ± 5.7 b 13 ± 2.6 b 199 ± 27 b 544 ± 51 c	290 ± 25 a 131 ± 19 a 1971 ± 25 c 45 ± 6.1 b 259 ± 41 b 21 ± 2.7 a 157 ± 34 a 8 ± 1.4 a 128 ± 21 a 32 ± 7.2 a 436 ± 64 a 3478 ± 270 b	81 ± 16 c 42 ± 3.3 c 362 ± 76 b 9 ± 1.0 d 147 ± 12 c 4 ± 0.1 d 25 ± 1.0 c ND 16 ± 1.2 b 14 ± 2.5 b 73 ± 4.0 c 774 ± 73 c	171 ± 25 b 85 ± 14 b 2975 ± 137 a 63 ± 9.3 a 576 ± 41 a 15 ± 2.2 b 50 ± 8.0 c ND 19 ± 3.1 b 39 ± 7.4 a 124 ± 13 c 4124 ± 165 a
EAA ^b^ Thr Val Met Ile Leu Phe Lys His Arg Total	31 ± 6.8 b 51 ± 6.6 b 7 ± 1.4 b 25 ± 4.9 b 48 ± 7.8 b 35 ± 6.3 c 29 ± 4.7 b 7 ± 0.8 c 20 ± 2.7 c 250 ± 43 c	72 ± 12 a 240 ± 46 a 20 ± 3.5 a 128 ± 30 a 115 ± 21 a 263 ± 28 a 91 ± 15 a 73 ± 6.8 b 283 ± 23 a 1284 ± 144 a	19 ± 1.1 b 43 ± 1.5 b ND 19 ± 1.5 b 21 ± 0.5 b 19 ± 2.3 c 14 ± 0.8 b 19 ± 1.5 c 10 ± 1.8 c 164 ± 9 c	22 ± 4.8 b 87 ± 13 b ND 35 ± 6.7 b 33 ± 2.8 b 162 ± 35 b 17 ± 3.7 b 105 ± 21 a 151 ± 20 b 611 ± 93 b
Total FAA ^c^	795 ± 90 b	4763 ± 407 a	938 ± 80 b	4736 ± 251 a

^a^ NEAA, non essential amino acid. ^b^ EAA, essential amino acid. ^c^ FAA, free amino acid. All values are the mean ± SD in triplicate. Means containing the same letter are not significantly different according to Tukey means test (*p* < 0.05) in each isoflavone.

## Data Availability

Not applicable.

## Supplementary Materials

The following are available online, Figures S1–S5: Quantitative analysis of soybean plant by amino acid analyzer, Figures S6 and S7 and Tables S1 and S2: Metabolomic analysis of soybean plant by GC/MS, Table S3: Nitrogen levels of control and ethylene treatment soybean leaves.
