# Supplementary material for: A Significant Change in Free Amino Acids of Soybean (Glycine max L. Merr) through Ethylene Application
Source: Molecules. 2021 Feb 20;26(4):1128. doi: 10.3390/molecules26041128 (PMC7924343; doi:10.3390/molecules26041128)
Supplement: Supplementary file 1 [file molecules-26-01128-s001.pdf]

Supplementary materials

A significant change in free amino acids of soybean (Glycine max L. Merr) through ethylene application

Yeong Jun Ban[1], Yeong Hun Song[1], Jeong Yoon Kim, Joon-Yung Cha, Imdad Ali, Baiseitova Aizhamal, Abdul Bari Shah, Woe-Yeon Kim, Ki Hun Park[*]

Division of Applied Life Science (BK21 plus), IALS, RILS, Gyeongsang National University, Jinju, 52828, Republic of Korea

► **Contents**

Figure S1-S5: Quantitative analysis of soybean plant by amino acid analyzer

Figure S6-7 and Table S1-2: Metabolomic analysis of soybean plant by GC/MS

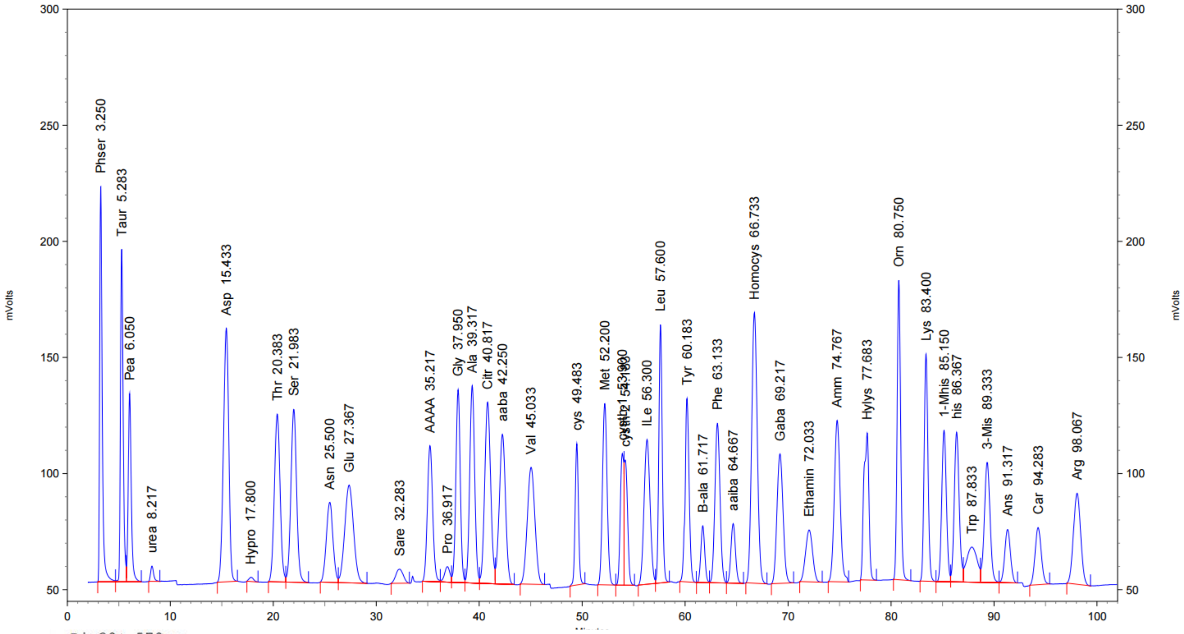

Bio30+ 570nm
Results

Pk #	Name	Retention Time	Area	Area Percent(%)	Conc.(nmol/40ul)
1	**Phser**	3.250	291772438	3.457	10.000 CAL
2	**Taur**	5.283	261068654	3.093	10.000 CAL
3	**Pea**	6.050	166225162	1.969	10.000 CAL
4	**urea**	8.217	13697082	0.162	10.000 CAL
5	**Asp**	15.433	391982580	4.644	10.000 CAL
6	**Hypro**	17.800	5611555	0.066	10.000 CAL
7	**Thr**	20.383	281632694	3.337	10.000 CAL
8	**Ser**	21.983	287777453	3.409	10.000 CAL
9	**Asn**	25.500	154664583	1.832	10.000 CAL
10	**Glu**	27.367	251002184	2.974	10.000 CAL
11	**Sare**	32.283	30528867	0.362	10.000 CAL
12	**AAAA**	35.217	209464605	2.482	10.000 CAL
13	**Pro**	36.917	23972201	0.284	10.000 CAL
14	**Gly**	37.950	248766302	2.947	10.000 CAL
15	**Ala**	39.317	286772753	3.397	10.000 CAL
16	**Citr**	40.817	317834349	3.765	10.000 CAL
17	**aaba**	42.250	274490765	3.252	10.000 CAL
18	**Val**	45.033	259856604	3.079	10.000 CAL
19	**cys**	49.483	136017394	1.611	5.000 CAL
20	**Met**	52.200	245421943	2.908	10.000 CAL
21	**cysth-1**	53.900	131953484	1.563	5.000 CAL
22	**cysth-2**	54.183	123369954	1.462	5.000 CAL
23	**ILe**	56.300	254095047	3.010	10.000 CAL
24	**Leu**	57.600	257001685	3.045	10.000 CAL
25	**Tyr**	60.183	212682710	2.520	10.000 CAL
26	**B-ala**	61.717	64765712	0.767	10.000 CAL
27	**Phe**	63.133	237956424	2.819	10.000 CAL
28	**aaiba**	64.667	78779836	0.933	10.000 CAL
29	**Homocys**	66.733	442197444	5.239	5.000 CAL
30	**Gaba**	69.217	229563348	2.720	10.000 CAL
31	**Ethamin**	72.033	106825744	1.266	10.000 CAL
32	**Amm**	74.767	273986380	3.246	10.000 CAL
33	**Hylys**	77.683	215455429	2.553	10.000 CAL
34	**Orn**	80.750	305023623	3.614	10.000 CAL
35	**Lys**	83.400	253457333	3.003	10.000 CAL
36	**1-Mhis**	85.150	205182390	2.431	10.000 CAL
37	**his**	86.367	216217507	2.562	10.000 CAL
38	**Trp**	87.833	108502990	1.285	10.000 CAL
39	**3-Mis**	89.333	201118970	2.383	10.000 CAL
40	**Ans**	91.317	88616888	1.050	5.000 CAL
41	**Car**	94.283	103126228	1.222	10.000 CAL
42	**Arg**	98.067	192438531	2.280	10.000 CAL
Totals			8440877825	100.000	395.000 CAL

Figure S1. Quantitative analysis of free amino acid standards by amino acid analyzer

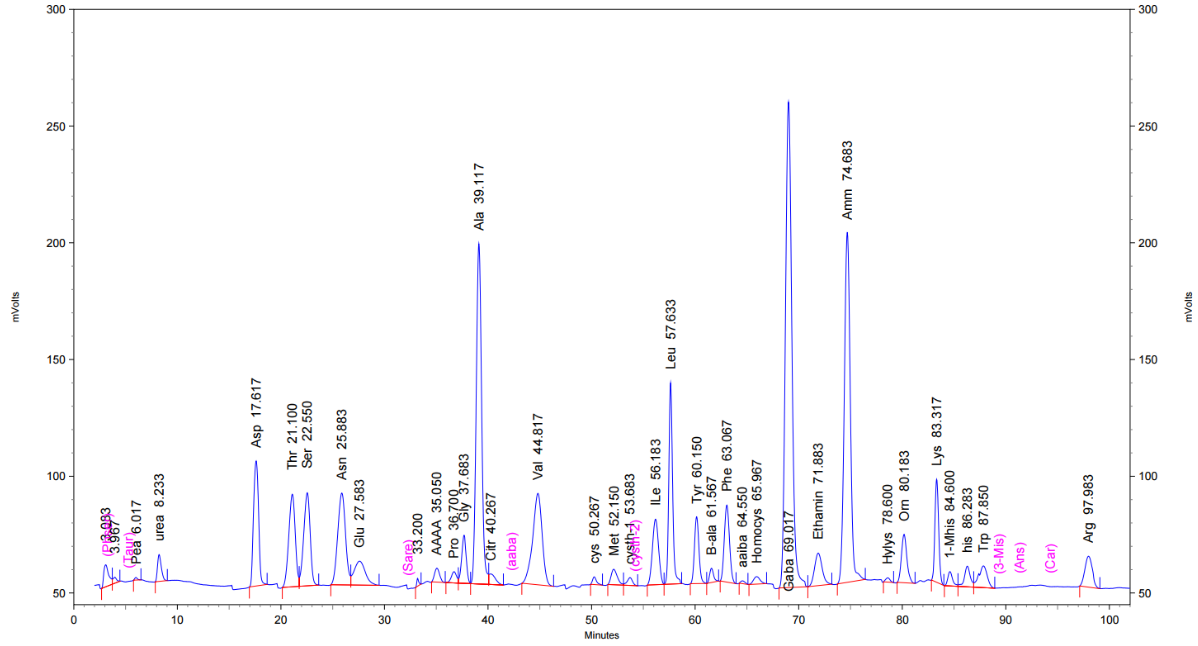

Bio30+ 570nm
Results

Pk #	Name	Retention Time	Area	Area Percent(%)	Conc.(nmol/40ul)
1		3.083	31333690	0.724	0.000
	Phser				0.000 BDL
2		3.967	6435485	0.149	0.000
	Taur				0.000 BDL
3	Pea	6.017	1920547	0.044	0.116
4	urea	8.233	29298460	0.677	21.390
	Hypro				0.000 BDL
5	Asp	17.617	181814314	4.204	324.000
6	Thr	21.100	172995236	4.000	3.894
7	Ser	22.550	173532225	4.012	4.062
8	Asn	25.883	205819960	4.759	13.308
9	Glu	27.583	76991652	1.780	3.067
	Sare				0.000 BDL
10		33.200	4283150	0.099	0.000
11	AAAA	35.050	20205528	0.467	0.965
12	Pro	36.700	16822407	0.389	7.017
13	Gly	37.683	62821609	1.452	2.525
14	Ala	39.117	528994361	12.231	18.446
15	Citr	40.267	19449587	0.450	0.612
	aaba				0.000 BDL
16	Val	44.817	247662625	5.726	9.531
17	cys	50.267	8621161	0.199	0.317
18	Met	52.150	27578126	0.638	1.124
19	cysth-1	53.683	11968544	0.277	0.454
	cysth-2				0.000 BDL
20	ILe	56.183	115400954	2.668	4.542
21	Leu	57.633	218552327	5.053	8.504
22	Tyr	60.150	85404295	1.975	4.016
23	B-ala	61.567	15945220	0.369	2.462
24	Phe	63.067	114469047	2.647	4.811
25	aaiba	64.550	3524954	0.081	0.447
26	Homocys	65.967	14088264	0.326	0.159
27	Gaba	69.017	891524050	20.612	38.836
28	Ethamin	71.883	70760794	1.636	6.624
29	Amm	74.683	601603655	13.909	21.957
30	Hylys	78.600	5327391	0.123	0.247
31	Orn	80.183	75736807	1.751	2.483
32	Lys	83.317	111364545	2.575	4.394
33	1-Mhis	84.600	19683915	0.455	0.959
34	his	86.283	34441490	0.796	1.593
35	Trp	87.850	52905566	1.223	4.876
	3-Mis				0.000 BDL
	Ans				0.000 BDL
	Car				0.000 BDL
36	Arg	97.983	65901479	1.524	3.425
Totals			4325183420	100.000	513.205

Figure S2. Quantitative analysis of soybean leaves (control) by amino acid analyzer

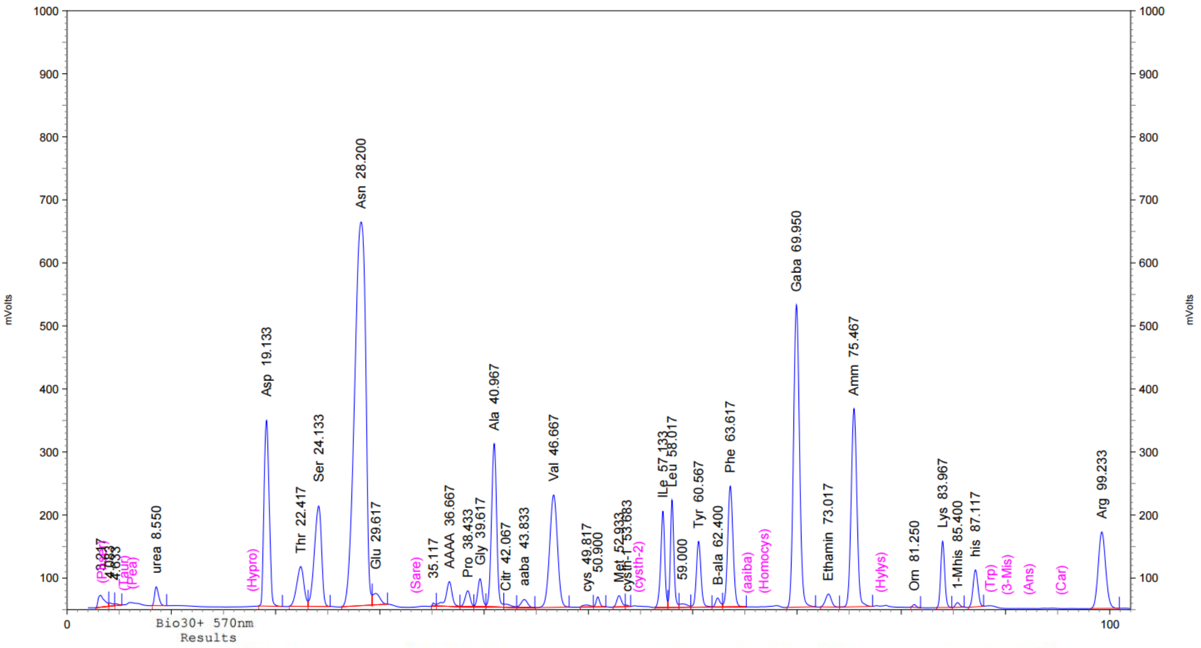

Pk #	Name	Retention Time	Area	Area Percent(%)	Conc.(nmol/40ul)
					0.000 BDL
1		3.217	79169285	0.513	0.000
	Phser				0.000 BDL
2		4.083	13575224	0.088	0.000
3		4.633	6242246	0.040	0.000
	Taur				0.000 BDL
	Pea				0.000 BDL
4	urea	8.550	79935468	0.518	56.149
					0.000 BDL
	Hypro				0.000 BDL
5	Asp	19.133	1018077227	6.599	4.943
6	Thr	22.417	305233452	1.978	10.452
7	Ser	24.133	759331482	4.922	4.062
8	Asn	28.200	4499268794	29.163	166.025
9	Glu	29.617	84667927	0.549	2.794
	Sare				0.000 BDL
10		35.117	9204943	0.060	0.000
11	AAAA	36.667	164818234	1.068	7.345
12	Pro	38.433	85831390	0.556	39.476
13	Gly	39.617	134900026	0.874	5.369
14	Ala	40.967	891118170	5.776	30.653
15	Citr	42.067	22686911	0.147	0.708
16	aaba	43.833	54694228	0.355	1.941
17	Val	46.667	936954845	6.073	34.916
18	cys	49.817	16038270	0.104	0.582
19		50.900	37173058	0.241	0.000
20	Met	52.933	56393235	0.366	2.349
21	cysth-1	53.683	4371753	0.028	0.166
	cysth-2				0.000 BDL
22	ILe	57.133	407815105	2.643	15.771
23	Leu	58.017	394158486	2.555	15.694
24		59.000	27564014	0.179	0.000
25	Tyr	60.567	321005492	2.081	14.176
26	B-ala	62.400	43767904	0.284	6.721
27	Phe	63.617	686261028	4.448	29.334
	aaiba				0.000 BDL
	Homocys				0.000 BDL
28	Gaba	69.950	1903492645	12.338	83.333
29	Ethamin	73.017	80840324	0.524	8.911
30	Amm	75.467	1203270353	7.799	46.542
	Hylys				0.000 BDL
31	Orn	81.250	9618092	0.062	0.327
32	Lys	83.967	278601868	1.806	11.423
33	1-Mhis	85.400	20775218	0.135	1.057
34	his	87.117	189194915	1.226	8.998
	Trp				0.000 BDL
	3-Mis				0.000 BDL
	Ans				0.000 BDL
	Car				0.000 BDL
35	Arg	99.233	601844258	3.901	30.680
Totals			15427895870	100.000	629.099

Figure S3. Quantitative analysis of ethylene treated soybean leaves (treatment) by amino acid analyzer

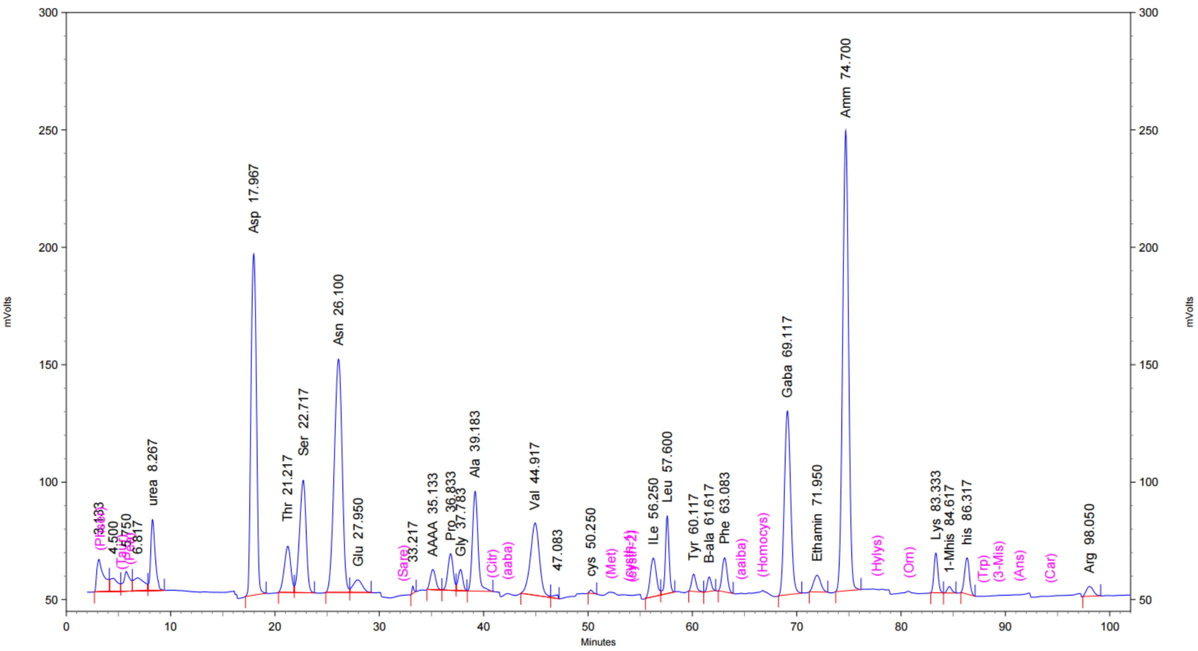

Bio30+ 570nm
Results

Pk #	Name	Retention Time	Area	Area Percent(%)	Conc.(nmol/40ul)
1		3.133	64717064	1.808	0.000
	Phser				0.000 BDL
2		4.500	29696138	0.830	0.000
	Taur				0.000 BDL
3		5.750	36229987	1.012	0.000
	Pea				0.000 BDL
4		6.817	39211166	1.095	0.000
5	urea	8.267	90956730	2.541	66.406
6	Asp	17.967	523692315	14.629	933.239
7	Thr	21.217	84933279	2.373	18.342
8	Ser	22.717	208572317	5.827	38.250
9	Asn	26.100	521219166	14.560	33.700
10	Glu	27.950	32689288	0.913	1.302
	Sarc				0.000 BDL
11		33.217	3624472	0.101	0.000
12	AAAA	35.133	30245884	0.845	1.444
13	Pro	36.833	55578301	1.553	23.184
14	Gly	37.783	27598545	0.771	1.109
15	Ala	39.183	156028342	4.359	5.441
	Citr				0.000 BDL
	aaba				0.000 BDL
16	Val	44.917	185073113	5.170	7.122
17		47.083	4753599	0.133	0.000
18	cys	50.250	2994779	0.084	0.110
	Met				0.000 BDL
	cysth-1				0.000 BDL
	cysth-2				0.000 BDL
19	ILe	56.250	66845150	1.867	2.631
20	Leu	57.600	81163941	2.267	3.158
21	Tyr	60.117	21854080	0.610	1.028
22	B-ala	61.617	16242723	0.454	2.508
23	Phe	63.083	48891540	1.366	2.055
	aaiba				0.000 BDL
	Homocys				0.000 BDL
24	Gaba	69.117	327334944	9.144	14.259
25	Ethamin	71.950	33885455	0.947	3.172
26	Amm	74.700	764519272	21.357	27.904
	Hylys				0.000 BDL
	Orn				0.000 BDL
27	Lys	83.333	44482328	1.243	1.755
28	1-Mhis	84.617	7988146	0.223	0.389
29	his	86.317	49398095	1.380	2.285
	Trp				0.000 BDL
	3-Mis				0.000 BDL
	Ans				0.000 BDL
	Car				0.000 BDL
30	Arg	98.050	19297262	0.539	1.003
Totals			3579717421	100.000	1135.204

Figure S4. Quantitative analysis of soybean stems (control) by amino acid analyzer

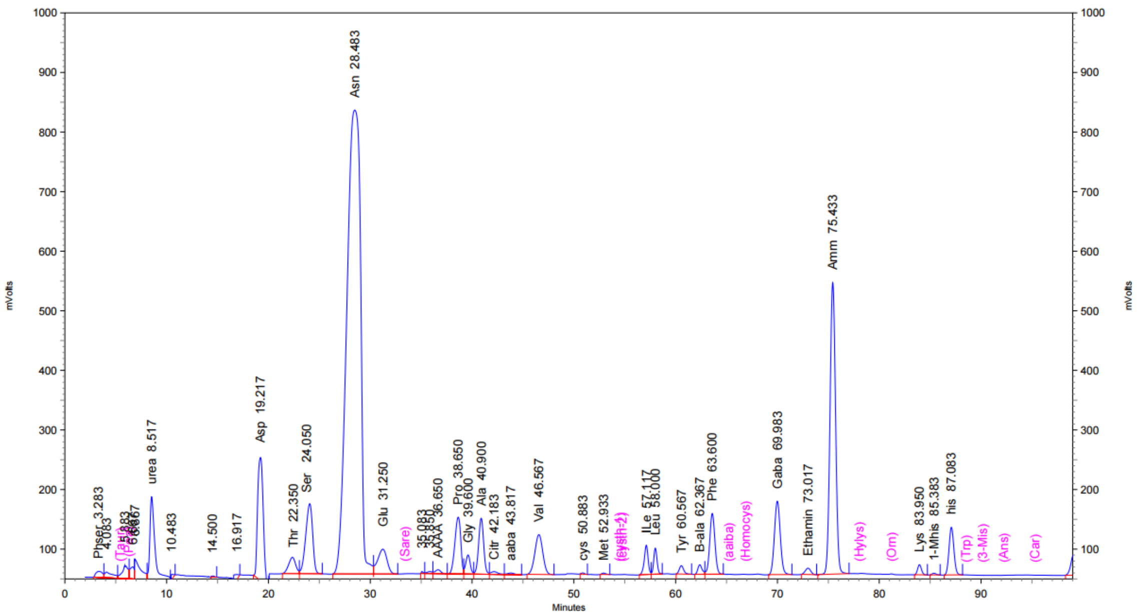

Bio30+ 570nm
Results

Pk #	Name	Retention Time	Area	Area Percent(%)	Conc.(nmol/40ul)
					0.000 BDL
1	Phser	3.283	45148492	0.305	1.529
2		4.083	52078509	0.352	0.000
	Taur				0.000 BDL
3		5.883	87829203	0.593	0.000
	Pea				0.000 BDL
4		6.667	56096332	0.379	0.000
5		6.867	136661230	0.923	0.000
6	urea	8.517	477871205	3.228	335.673
7		10.483	11994039	0.081	0.000
8		14.500	2895054	0.020	0.000
					0.000 BDL
9		16.917	31617603	0.214	0.000
10	Asp	19.217	785149117	5.304	73.000
11	Thr	22.350	131415795	0.888	4.500
12	Ser	24.050	556026170	3.756	100.000
13	Asn	28.483	7289977661	49.244	2693.003
14	Glu	31.250	267270937	1.805	0.000
	Sare				0.000 BDL
15		35.083	4298149	0.029	0.000
16		35.850	11881100	0.080	0.000
17	AAAA	36.650	26058242	0.176	1.161
18	Pro	38.650	383555820	2.591	176.409
19	Gly	39.600	98737715	0.667	3.930
20	Ala	40.900	325333635	2.198	11.191
21	Citr	42.183	22131740	0.150	0.690
22	aaba	43.817	12178420	0.082	0.432
23	Val	46.567	361670598	2.443	13.478
24	cys	50.883	3989986	0.027	0.145
25	Met	52.933	5800059	0.039	0.242
	cysth-1				0.000 BDL
	cysth-2				0.000 BDL
26	ILe	57.117	129342115	0.874	13.529
27	Leu	58.000	98931377	0.668	3.939
28	Tyr	60.567	42296450	0.286	1.868
29	B-ala	62.367	47059711	0.318	7.227
30	Phe	63.600	362390315	2.448	15.490
	aaiba				0.000 BDL
	Homocys				0.000 BDL
31	Gaba	69.983	491739463	3.322	21.528
32	Ethamin	73.017	39401024	0.266	4.343
33	Amm	75.433	1860351516	12.567	71.958
	Hylys				0.000 BDL
	Orn				0.000 BDL
34	Lys	83.950	44676813	0.302	1.832
35	1-Mhis	85.383	7086447	0.048	0.361
36	his	87.083	265347989	1.792	12.619
	Trp				0.000 BDL
	3-Mis				0.000 BDL
	Ans				0.000 BDL
	Car				0.000 BDL
37	Arg	99.250	227462800	1.537	11.595
Totals			14803752831	100.000	976.144

Figure S5. Quantitative analysis of ethylene treated soybean stems (treatment) by amino acid analyzer

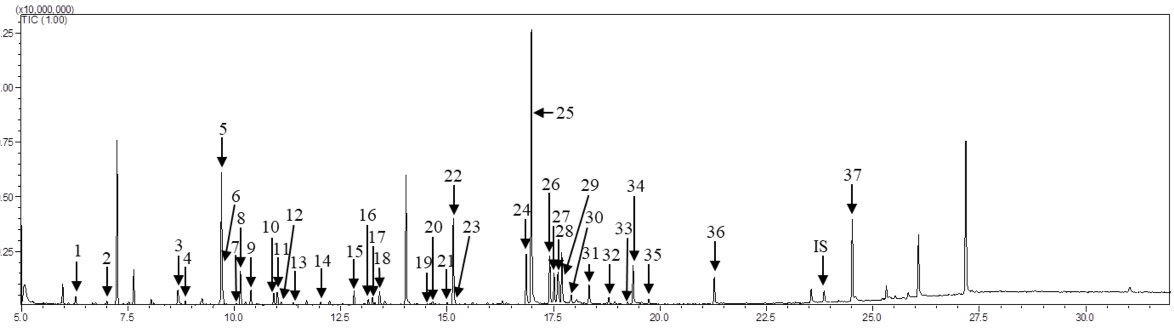

1, lactic acid; 2, alanine; 3, malonic acid; 4, valine; 5, phosphoric acid; 6, glycerol; 7, isoleucine; 8, proline; 9, succinic acid; 10, fumaric acid; 11, serine; 12, nonanoic acid; 13, threonine; 14, beta-alanine; 15, malic acid; 16, aspartic acid; 17, oxoproline; 18, 4-aminobutanoic acid; 19, glutamic acid; 20, phenylalnine; 21, arabinose; 22, asparagine; 23, lyxose; 24, citric acid; 25, pinitol; 26, fructose; 27, fructose; 28, galactose; 29, mannose; 30, glucose; 31, inositol; 32, gluconic acid; 33, galactaric acid; 34, palmitic acid; 35, myo-inositol; 36, stearic acid; 37, sucrose

Figure S6. Representative GC/MS chromatogram of soybean stems

Table S1. Identified metabolites of soybean leaves contributing to the GC/MS chromatogram (Figure S6) of the dataset analyzed by GC/MS

No	Identify	RT (min)[a]	RI[b]	VIP[c]	*p*-value[d]	No	Identify	RT (min)[a]	RI[b]	VIP[c]	*p*-value[d]
1	lactic acid	6.27	1053	0.19	0.045	19	phenylalnine	14.66	1622	0.23	0.001
2	alanine	7.00	1097	0.20	6.74E-04	20	arabinose	14.99	1649	0.03	0.511
3	malonic acid	8.67	1198	0.25	0.253	21	asparagine	15.14	1661	1.39	0.002
4	valine	8.85	1209	0.33	6.98E-05	22	lyxose	15.18	1664	0.06	0.865
5	phosphoric acid	9.70	1264	1.66	6.72E-04	23	citric acid	16.86	1807	1.33	0.002
6	glycerol	9.73	1266	0.17	0.653	24	pinitol	16.98	1818	3.11	0.122
7	isoleucine	10.05	1286	0.14	0.006	25	fructose	17.41	1856	1.40	0.147
8	proline	10.14	1292	0.13	0.589	26	fructose	17.51	1866	1.07	0.123
9	succinic acid	10.39	1308	0.04	0.110	27	galactose	17.62	1876	0.38	0.068
10	fumaric acid	10.92	1345	0.09	0.044	28	mannose	17.69	1882	2.81	0.012
11	serine	11.03	1352	0.40	2.25E-04	29	glucose	17.91	1902	0.59	0.025
12	threonine	11.39	1377	0.09	6.26E-04	30	inositol	18.33	1942	0.18	0.472
13	malic acid	12.81	1479	0.29	0.310	31	gluconic acid	18.80	1987	0.48	0.010
14	aspartic acid	13.25	1512	0.22	4.00E-05	32	galactaric acid	19.24	2030	0.08	3.75E-04
15	oxoproline	13.32	1517	0.03	9.74E-04	33	palmitic acid	19.37	2043	0.23	0.197
16	GABA	13.41	1525	0.80	0.002	34	myo-inositol	19.73	2079	0.06	0.319
17	threonic acid	13.72	1549	0.01	0.842	35	stearic acid	21.27	2239	0.12	0.276
18	glutamic acid	14.53	1611	0.06	1.39E-05	36	sucrose	24.51	2593	3.36	0.002

[a]RT is retention time.
[b]RI is retention index calculated with *n*-alkanes.
[c]VIP is variable importance in the projection values were determined by PLS-DA.
[d]*p*-values were analyzed by ANOVA with Duncan's test.

Table S2. Identified metabolites of soybean stems contributing to the GC/MS chromatogram (Figure S7) of the dataset analyzed by GC/MS

No	Identify	RT (min)[a]	RI[b]	VIP[c]	*p*-value[d]	No	Identify	RT (min)[a]	RI[b]	VIP[c]	*p*-value[d]
1	lactic acid	6.27	1053	0.41	0.073	20	phenylalnine	14.66	1622	0.16	0.133
2	alanine	7.01	1097	0.07	0.048	21	arabinose	15.00	1649	0.13	0.062
3	malonic acid	8.68	1198	0.56	0.151	22	asparagine	15.14	1661	2.92	0.378
4	valine	8.85	1209	0.05	0.678	23	lyxose	15.17	1663	0.06	0.775
5	phosphoric acid	9.70	1264	2.32	0.001	24	citric acid	16.85	1807	1.10	0.621
6	glycerol	9.73	1266	0.32	0.021	25	pinitol	16.98	1817	1.14	0.787
7	isoleucine	10.05	1286	0.03	0.233	26	fructose	17.41	1856	1.76	0.334
8	proline	10.14	1292	1.35	0.085	27	fructose	17.51	1865	0.78	0.564
9	succinic acid	10.39	1309	0.79	0.095	28	galactose	17.60	1874	2.48	0.183
10	fumaric acid	10.93	1345	0.83	0.002	29	mannose	17.69	1882	1.72	0.507
11	serine	11.03	1352	0.10	0.520	30	glucose	17.91	1901	0.46	0.414
12	nonanoic acid	11.10	1357	0.02	0.557	31	inositol	18.33	1942	0.27	0.417
13	threonine	11.39	1377	0.03	0.316	32	gluconic acid	18.79	1987	0.10	0.370
14	beta-alanine	12.03	1422	0.06	0.176	33	galactaric acid	19.23	2029	0.13	1.65E-04
15	malic acid	12.81	1479	0.80	0.006	34	palmitic acid	19.36	2043	0.23	0.292
16	aspartic acid	13.25	1512	0.08	0.572	35	myo-inositol	19.73	2079	0.02	0.925
17	oxoproline	13.30	1516	0.05	0.326	36	stearic acid	21.27	2239	0.15	0.311
18	4-aminobutanoic acid	13.41	1525	0.22	0.450	37	sucrose	24.51	2593	2.26	0.697
19	glutamic acid	14.53	1611	0.06	0.024						

[a]RT is retention time.
[b]RI is retention index calculated with *n*-alkanes.
[c]VIP is variable importance in the projection values were determined by PLS-DA.
[d]*p*-values were analyzed by ANOVA with Duncan's test.

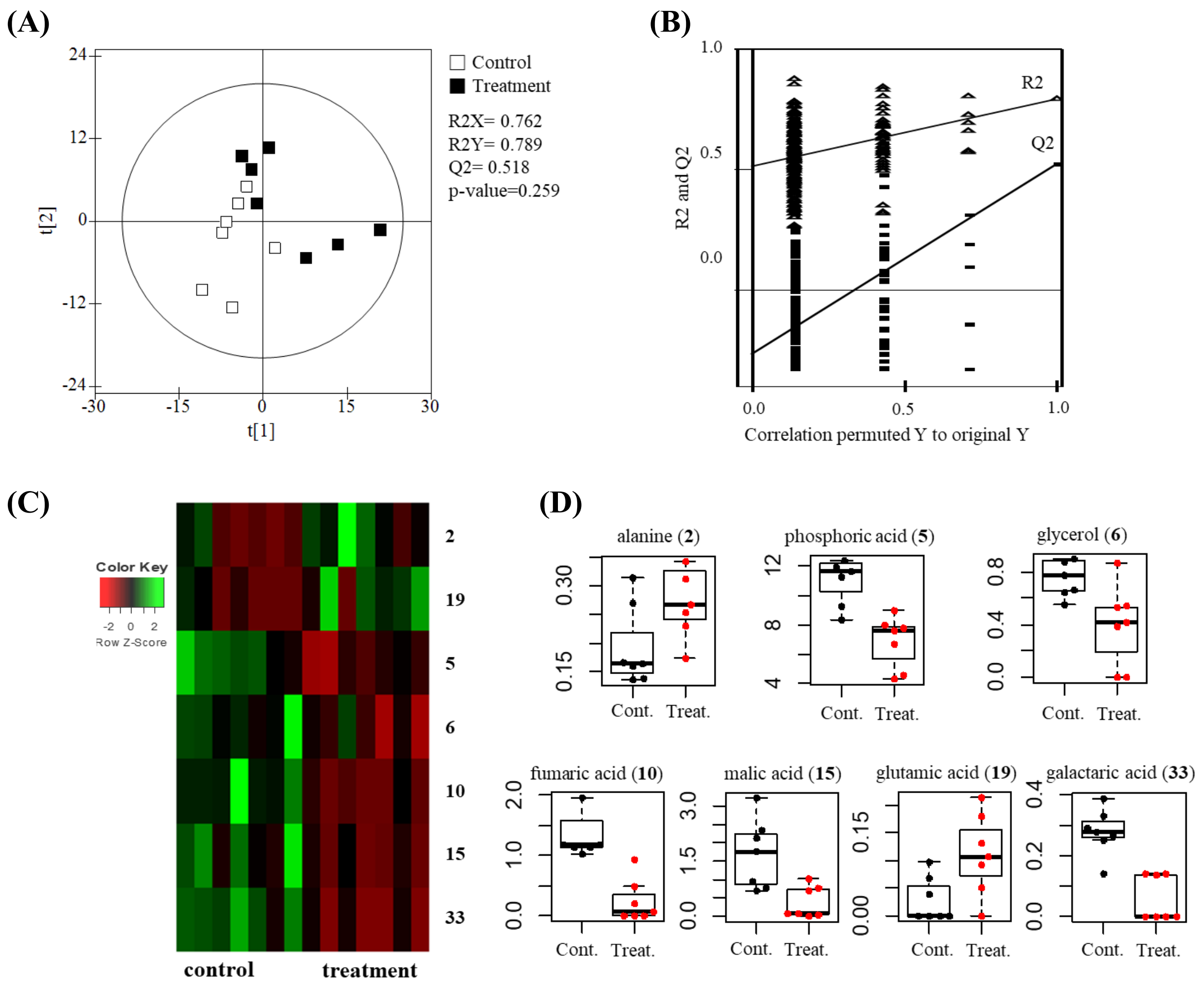

Figure S7. Metabolomic analysis of soybean stems by GC/MS. (A) PLS-DA plots of soybean stems untreated (control) and treated ethylene (treatment). (B) Performance of the permutation tests validated from the PLS-DA model. (C) Heatmap for the 7 metabolites (p < 0.05) in soybean stems between control and treatment. (D) Boxplot of representative metabolites in soybean stems.
